# Baseline atrial volume indices and major adverse cardiac events following thoracic radiotherapy

**DOI:** 10.3389/fcvm.2025.1560922

**Published:** 2025-06-03

**Authors:** Edmund M. Qiao, John He, Katrina D. Silos, Jordan O. Gasho, Patrick Belen, Danielle S. Bitterman, Elizabeth McKenzie, Jennifer Steers, Christian Guthier, Anju Nohria, Michael T. Lu, Hugo J. W. L. Aerts, Andriana P. Nikolova, Raymond H. Mak, Katelyn M. Atkins

**Affiliations:** ^1^ Department of Radiation Medicine and Applied Sciences, University of California San Diego, La Jolla, CA, United States; ^2^ Department of Radiation Oncology, Brigham and Women’s Hospital/Dana-Farber Cancer Institute, Boston, MA, United States; ^3^ Department of Radiation Oncology, Cedars-Sinai Medical Center, Los Angeles, CA, United States; ^4^ Artificial Intelligence in Medicine (AIM) Program, Mass General Brigham and Harvard Medical School, Boston, MA, United States; ^5^ Department of Cardiovascular Medicine, Brigham and Women’s Hospital/Dana-Farber Cancer Institute, Boston, MA, United States; ^6^ Department of Radiology, Cardiovascular Imaging Research Center, Massachusetts General Hospital and Harvard Medical School, Boston, MA, United States; ^7^ Radiology and Nuclear Medicine, GROW & CARIM Maastricht University, Maastricht, Netherlands; ^8^ Department of Cardiology, Cedars-Sinai Medical Center, Los Angeles, CA, United States

**Keywords:** oncology, radiotherapy, lung, breast, major adverse cardiac events, atrial volume, radiation oncology

## Abstract

**Introduction:**

Patients receiving thoracic radiotherapy (RT) have an increased risk of major adverse cardiac events (MACE) posttreatment. We utilized machine learning (ML) to discover novel predictors of MACE and validated them on an external cohort.

**Methods:**

This multi-institutional retrospective study included 984 patients [*n* = 803 non-small cell lung cancer (NSCLC), *n* = 181 breast cancer] treated with radiotherapy. Extreme gradient boosting was utilized to discover novel clinical, dosimetric, and anatomical features (CT-based cardiac substructure segmentations) associated with MACE in a cohort of locally advanced NSCLC patients. Fine–Gray regression was performed with non-cardiac death as a competing risk. External validation was performed utilizing independent cohorts of NSCLC or breast cancer patients.

**Results:**

In the discovery dataset (*n* = 701), 70 patients experienced MACE. ML modeling (training AUC, 0.68; testing AUC, 0.71) identified right and left atrial volume indices (RAVI and LAVI, respectively) as top predictors. After adjusting for baseline cardiovascular risk and known radiotherapy predictive factors, RAVI was associated with an increased risk of MACE [subdistribution hazard ratio (sHR) 1.02/unit, 95% confidence interval (CI): 1.00–1.04; *p* = 0.03]. In the validation cohorts (*n* = 102 NSCLC; *n* = 181 breast cancer), RAVI was associated with an increased risk of MACE (NSCLC: sHR 1.05, 95% CI: 1.001–1.106, *p* = 0.04; breast cancer: sHR 1.06, 95% CI: 1.01–1.11, *p* = 0.03). Similar findings were found for LAVI.

**Discussion:**

ML modeling identified right and left atrial enlargement as novel radiographic predictors for increased risk of MACE following chest radiotherapy, which was validated in independent breast and lung cancer datasets. Given that echocardiography studies have demonstrated the prognostic utility of atrial volume indices across cardiovascular risk groups, these findings warrant further study to identify additional strategies for upfront cardiovascular risk profiling.

## Introduction

Radiotherapy (RT) forms the cornerstone of definitive treatment for many thoracic and chest malignancies (1–4); however, RT-associated cardiac toxicity remains a significant risk (2–5). Among primary chest malignancies, the highest rates of major adverse cardiac events (MACE) are observed in patients with lung cancer (6). For non-small cell lung cancer (NSCLC) patients undergoing definitive chemoradiation, RT-associated MACE occur early (median onset within 2 years) and are associated with increased mortality (3, 5).

Several studies have identified RT dose and volume metrics associated with increased risk of MACE following RT. These studies specifically outline cardiac substructure dose constraints for the left heart, left coronary arteries—left anterior descending (LAD) coronary artery and left circumflex (LCx) coronary artery—and left ventricle (LV) (2, 7). NSCLC patients are enriched for traditional patient-level cardiovascular risk factors, such as older age, smoking, and coronary heart disease (CHD) (8, 9). Indeed, 25%–40% of lung cancer patients have concomitant CHD (10, 11). Given elevated baseline cardiovascular risks, combined with known cancer therapy-related cardiovascular toxicities, NSCLC patients have an unmet clinical need for improved baseline cardiovascular risk stratification for MACE following chest radiotherapy.

Point-of-care interactions between patients and radiation oncologists represent informative time points for cardiovascular risk stratification. Mapping the contribution of predictors remains challenging because of complex interactions between the vast number of RT variables and patient-level cardiovascular risk factors. Tree-based machine learning (ML) captures imperceptible patterns from diverse inputs while limiting multicollinearity from high-dimensional data and offering reduced bias (omitted variable, confirmation, etc.). Given its continued applications (12–15), we utilized tree-based ML to identify MACE predictors from an expanded pool of features, including baseline cardiac health, cardiac substructure anatomy from volumetric CT segmentations, cancer-specific variables, and RT covariates including cardiac substructure dosimetry. These were identified in a cohort of locally advanced NSCLC patients. To evaluate generalizability, we validated a mixed cohort of patients who received chest RT for NSCLC or breast cancer at an external institution.

## Materials and methods

### Patient cohorts and treatment

This multi-institutional retrospective study included patients with locally advanced NSCLC or breast cancer treated with chest radiotherapy. ML modeling utilized 701 NSCLC patients treated between December 2003 and January 2014 at Brigham and Women's Hospital and Dana-Farber Cancer Institute, Boston, Massachusetts, denoted as the *discovery* dataset. External validation was performed on 273 patients treated between August 2005 and August 2021 at Cedars-Sinai Medical Center, Los Angeles, California, denoted as the *validation* dataset. To explore generalizability, the validation dataset included 181 breast cancer patients (16) and 102 NSCLC patients (17). Radiotherapy was delivered using 3D conformal RT (3D-CRT) or intensity-modulated RT (IMRT), excluding stereotactic body radiotherapy. For NSCLC patients, treatments were delivered free-breathing, typically based on internal-target volumes generated using four-dimensional CT scans (breath-hold or phase-based gating was not used). For the breast cancer validation cohort, deep-inspiration breath hold (typically for left-sided cancer) was utilized beginning in 2012. Other radiation planning specifics are previously described (2, 16, 17).

### Clinical and radiotherapy features

Baseline clinical variables were curated from an in-depth medical record review, including CHD, congestive heart failure (CHF), arrhythmia, statin use, and cardiac risk factors (hyperlipidemia, hypertension (HTN), smoking, diabetes mellitus). CHD included coronary artery disease (CAD), heart failure (HF), or a CHD risk equivalent (peripheral vascular disease or stroke) (3). Cancer treatment-specific variables included chemotherapy, surgery, and RT. Cardiac substructure variables were generated (for the discovery cohort) by manual delineation of cardiac chambers and coronary arteries on non-electrocardiogram-gated radiotherapy planning CTs, as previously described (2, 17). For the validation cohort, an automated deep learning algorithm segmented cardiac substructures and was manually verified (CG) (18). RT dose was converted to an equivalent dose in 2 Gy fractions for tumor and normal tissue. The *α*/*β* ratios utilized for normal tissue (esophagus, lung, heart, and cardiac chambers) and NSCLC tumor were 3 and 10, respectively. Cardiac chamber volumes were indexed to body surface area (BSA), including right atrial volume index (RAVI) and left atrial volume index (LAVI). RT dosimetric variables, including mean (Gy), maximum (Gy), and volume (percent) receiving specific (X) gray dose [VX Gy (5 Gy increments)] were calculated for the lungs, esophagus, heart, and cardiac substructures (chambers and coronaries). For the training and test datasets, the primary endpoint was MACE (unstable angina, HF hospitalization or urgent visit, myocardial infarction, coronary revascularization, and cardiac death) following initiation of RT or after 30 days postoperatively, if applicable (19). For patients with preexisting cardiac comorbidity, MACE was recorded if the cardiac event was either greater in severity compared with the 6 months prior to radiotherapy or of a different MACE category (3, 20). Comprehensive, manual medical record review delineated cardiac events, as previously described (3).

### Statistical analysis

Continuous variables were compared using the Wilcoxon rank sum test and categorical variables using the chi-square or Fisher exact test. Follow-up was calculated from RT start using the reverse Kaplan–Meier method. Extreme gradient boosting (XGBoost) (21) identified covariates related to developing MACE within the discovery cohort. The small proportion of missing data was binned into categorical unknown columns. The discovery dataset was split into training (75%) and test (25%) data (Supplementary Table S1). The training data constructed models, and the test dataset assessed model performance. XGBoost hyperparameters were bootstrap-tuned with a 50-round grid search during model training. The area under the receiver operator characteristic curve (AUC) evaluated model performance, and total gain ranked feature importance.

The Cedars-Sinai validation dataset was excluded from ML modeling and internal validation. Internal validation was performed on the top 15 ML-identified features utilizing the entire discovery dataset. Univariable and multivariable Fine–Gray regression models were utilized to evaluate the relationship between MACE and top predictors, with non-cardiac death as a competing risk. The multivariable analysis included ML-identified features that were significant in univariate analysis and variables with known prognostic value. Variance inflation factor and tolerance were used to assess multicollinearity. Given the multicollinearity between heart volume variables, when testing the multivariable association between MACE and a given substructure's volume, models were limited to a single cardiac volumetric parameter. External validation of the most predictive ML-identified features from the discovery dataset was performed using multivariable Fine–Gray regression on the Cedars-Sinai validation dataset. Analysis was performed utilizing R v4.2.2. and Stata SE, v17.0 (StataCorp LLC).

## Results

### Baseline characteristics

In the discovery cohort (*n* = 701), 345 (49.2%) were women, 252 (36.0%) had CHD, and 623 (88.9%) had clinical Stage III NSCLC. The median RT dose was 66.0 Gy (IQR, 56.0–66.0), with 539 (76.9%) receiving 3D-CRT. In the validation cohort of NSCLC patients (*n* = 102), 56 (54.9%) were women, 32 (31.4%) had CHD, and 74 (72.5%) had clinical Stage III disease. The median RT dose was 60.0 Gy (IQR, 55.8–60.0), with 20 (19.6%) receiving 3D-CRT. NSCLC patients from the validation cohort were generally older and had a lower prevalence of Stage III disease, lower 3D-CRT usage, and lower prescribed RT dose. Breast cancer patients from the validation cohort generally had lower rates of smoking and cardiac comorbidities (Table 1; Supplementary Table S2).

**Table 1:** Patient characteristics across discovery and external validation cohorts.

Characteristic	Discovery NSCLC cohort (*n* = 701)	Validation cohort			
		NSCLC (*n* = 102)	*p* (vs. discovery)	Breast cancer (*n* = 181)	*p* (vs. discovery)
Age median (IQR, years)	65 (57, 73)	71 (64, 77)	<0.0001	63 (53, 72)	0.073
Female sex	345 (49.2%)	56 (54.9%)	0.46	181 (100%)	<0.001
Tobacco					
Never	56 (8.0%)	24 (23.5%)		114 (63.3%)	
Current	279 (39.8%)	11 (10.8%)		6 (3.3%)	
Former	366 (52.2%)	67 (65.7%)		60 (33.3%)	
Unknown	0 (0.0%)	0 (0.0%)	<0.001	1 (0.6%)	<0.001
Medical history					
HTN	362 (51.6%)	66 (64.7%)	0.25	77 (42.5%)	0.030
HLD	341 (48.6%)	56 (54.9%)	0.25	108 (59.7%)	0.055
DM	97 (13.8%)	30 (29.4%)	<0.001	25 (13.8%)	1.0
Stroke	13 (1.9%)	7 (6.9%)	0.008	1 (0.6%)	0.32
CAD	202 (28.8%)	32 (31.4%)	0.78	9 (5.0%)	<0.001
CHF	58 (8.3%)	8 (7.8%)	1.0	3 (1.7%)	0.001
Any CHD^b^	252 (36.0%)	32 (31.4%)	0.44	97 (53.6%)	<0.001
NSCLC clinical stage					
II III Unknown	78 (11.1%) 623 (88.9%) 0 (0.0%)	I–II: 19 (18.6%) III: 74 (72.5%) IV: 8 (7.8%) Unknown: 1 (0.9%)	0.033	^a^I-II: 137 (76.0%) III/IV: 44 (24.3%) Unknown: 0 (0.0%)	NA
Tumor laterality					
Right	392 (55.9%)	68 (66.7%)		84 (46.4%)	
Left	263 (37.5%)	34 (33.3%)		97 (53.6%)	
NA/unknown	46 (6.6%)	0 (0.0%)	0.23	0 (0.0%)	0.002
Treatment					
Definitive CRT	405 (57.8%)	63 (61.8%)		BCT:	
RT alone	56 (8.0%)	4 (3.9%)		122 (67.4%)	
Neoadjuvant	154 (22.0%)	4 (3.9%)		Mastectomy:	
Adjuvant	86 (12.3%)	31 (30.4%)	<0.001	59 (32.6%)	NA
RT technique					
3D-CRT	539 (76.9%)	20 (19.6%)		175 (96.7%)	
IMRT	162 (23.1%)	82 (80.4%)	<0.001	6 (3.3%)	NA
RT dose					
Median (IQR, Gy)	66.0 (56.0, 66.0)	60.0 (55.8, 60.0)	<0.001	50.0 (42.7, 50.4)	NA

Individual values are listed to represent *n* (%) unless otherwise specified as median (IQR).

IQR, interquartile range; HTN, hypertension; HLD, hyperlipidemia; DM, diabetes mellitus; CAD, coronary artery disease; CHF, congestive heart failure; CHD, coronary heart disease; NSCLC, non-small cell lung cancer; CRT, chemoradiotherapy; BCT, breast-conserving treatment; Gy, Gray; 3D-CRT, three-dimensional conformal radiation therapy; IMRT, intensity-modulated radiation therapy; NA, not applicable.

^a^ Pathological breast cancer stage.

^b^ CHD includes CAD, CHF, or CHD risk equivalent (stroke, peripheral artery disease).

### ML identification of novel predictive features of MACE

**Figure 1:**
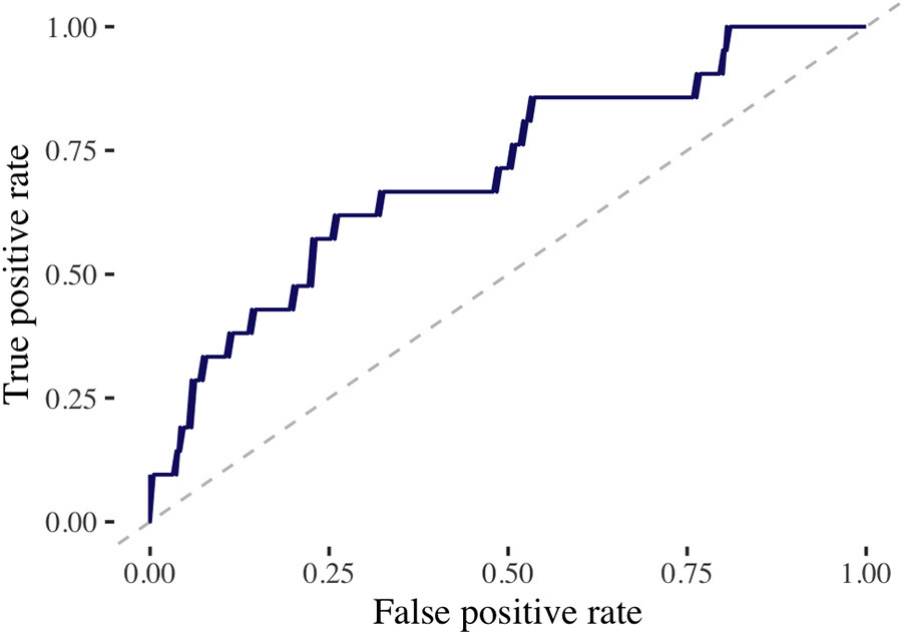
Performance of machine learning model on test dataset. This figure demonstrates the receiving operating characteristic curve for the extreme gradient boosting model to predict MACE in the test dataset utilizing the discovery cohort. The area under the curve (AUC) assesses the model’s accuracy. AUC, 0.71.

**Figure 2:**
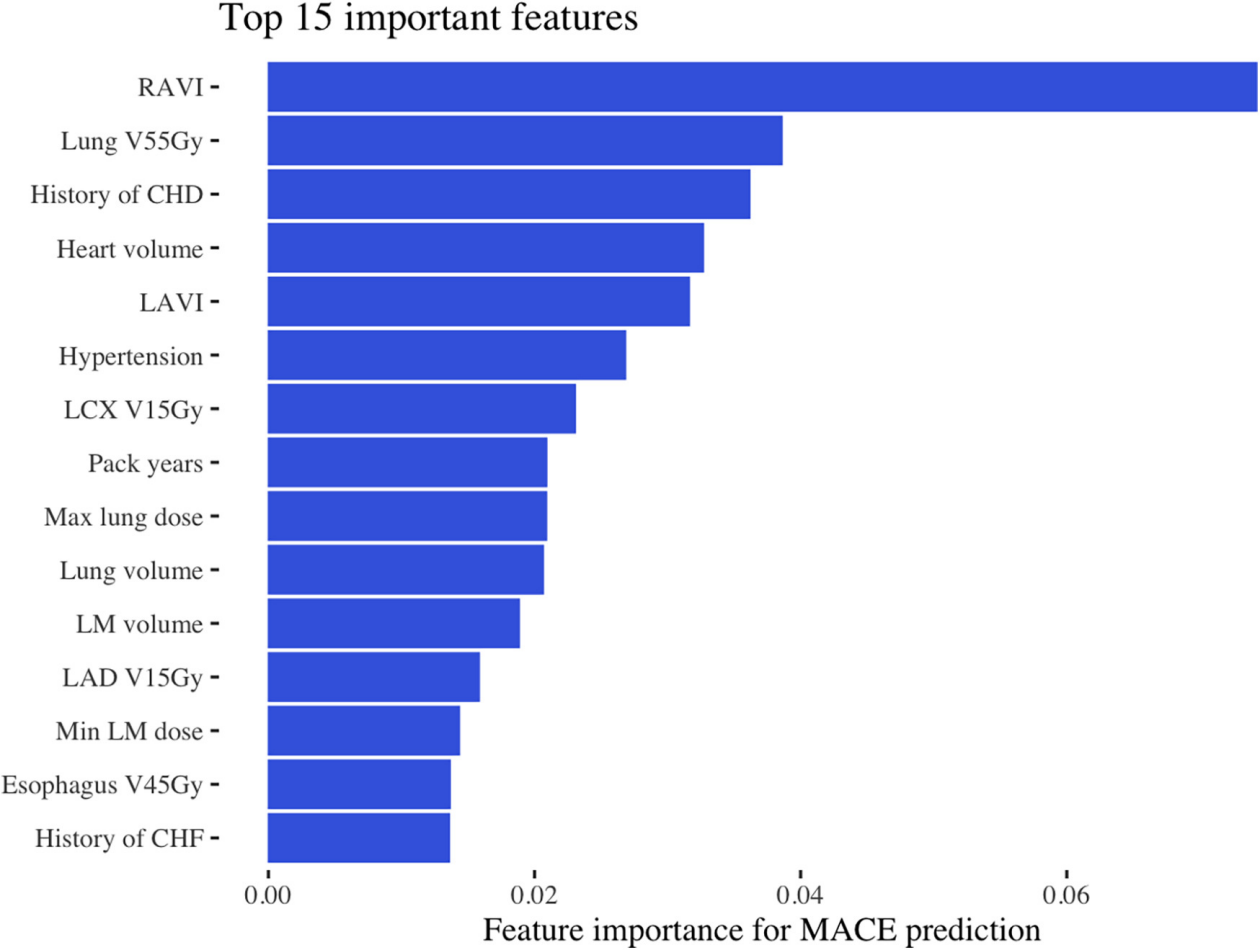
Most important features for MACE prediction. This figure shows the top 15 most important features identified by the machine learning model in the discovery dataset, ranked by total gain. RAVI, right atrial volume index; Gy, Gray; V55Gy, volume receiving 55 Gray; CHD, coronary heart disease LAVI, left atrial volume index; LCx, left circumflex artery; V15Gy, volume receiving 15 Gray; D_min_, dose minimum; V45Gy, volume receiving 45 Gray; CHF, congestive heart failure.

In the discovery cohort, with over a median follow-up of 5.2 years (IQR, 3.4–7.8 years), there were 70 cases of MACE (10%) with a median time to MACE of 1.6 years (IQR, 0.5–2.8 years). The final model included 27 baseline characteristics, 164 cancer-specific or treatment-related variables, and 197 dose/volume variables (Supplementary Appendix). The training AUC for MACE was 0.68, and the testing AUC was 0.71 (Figure 1). The top predictive feature was RAVI, followed by lung V55Gy and CHD (Figure 2). Additional important predictors included cardiovascular risk factors (hypertension, CHF), RT dose (LCx V15Gy, LADV15Gy, lung V55Gy), and BSA-normalized cardiac substructure volumes (RAVI, LAVI, total heart volume, left main coronary artery volume). Multiple ML-identified features have been previously reported as predictors of MACE and/or cardiac toxicity (CHD, HTN, LCxV15Gy, and LADV15Gy). Novel features included RAVI, LAVI, and lung V55Gy.

### RAVI and LAVI predict MACE in the internal competing risk regression model

**Figure 3:**
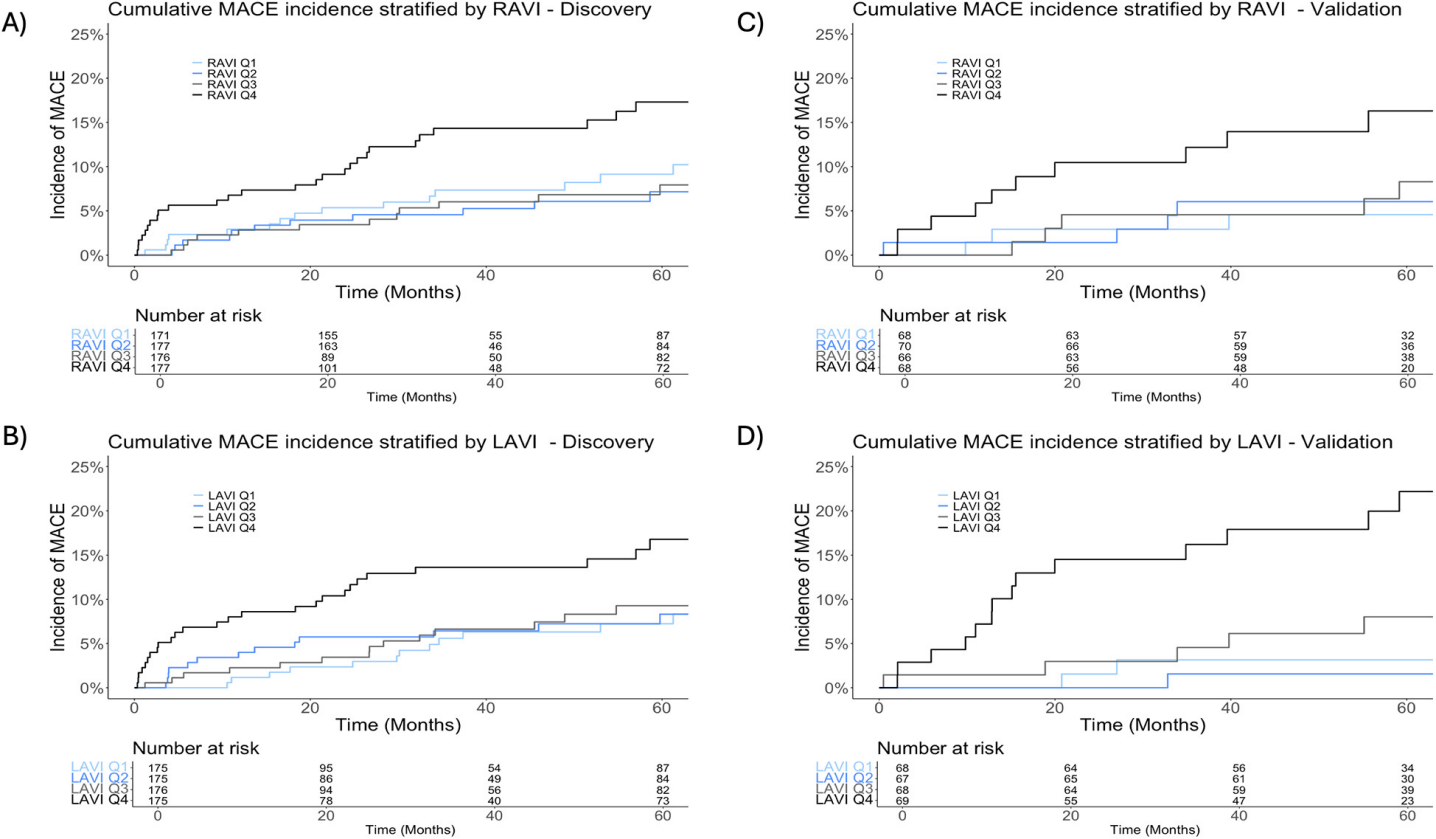
Cumulative incidence of major adverse cardiac events (MACE). Panels stratified by quartiles of right atrial volume indexed to body surface area (RAVI) and left atrial volume indexed to body surface area (LAVI) in the total (*n* = 701) discovery dataset **(A,B)** and the validation dataset (*n* = 283) **(C,D)**.

On univariate analysis, each unit (ml/m^2^) increase in RAVI was associated with a 2% increased risk of MACE [subdistribution hazard ratio (sHR) 1.02, 95% confidence interval (CI): 1.01–1.04; *p* = 0.001]. Additionally, each unit (ml/m^2^) increase in LAVI was associated with a 2% increased risk of MACE (sHR 1.02, 95% CI: 1.01–1.03; *p* = 0.001, Table 2). Lung V55Gy, lung volume and maximum dose, esophagus V45Gy, left main (LM) coronary artery minimum dose, and smoking pack-years were not significantly associated with MACE on univariable analysis. Notably, total heart volume and LM volume were significantly associated with MACE on univariable analysis. To limit multiple testing and collinearity, we focused exploration on RAVI and LAVI given RAVI's importance on ML analysis and the known impact of elevated atrial chamber volumes as clinical indicators for cardiac disease (22, 23). The median RAVI and LAVI values in the discovery cohort were 50.9 ml/m^2^ (IQR, 42.1–62.8 ml/m^2^) and 47.9 ml/m^2^ (IQR, 40.3–56.5 ml/m^2^), respectively. The cumulative incidence of MACE appeared similar for the first three RAVI quartiles but significantly increased for the highest quartile (RAVI ≥ 62.8 ml/m^2^; *p* = 0.02). LAVI showed similar results; the highest quartile (LAVI ≥ 56.5 ml/m^2^) trended toward significance (*p* = 0.052) (Figures 3A,B).

**Table 2:** Fine–Gray regression model to predict MACE in the discovery cohort (*n* = 701).

Covariable	Univariable		Multivariable	
	HR (95% CI)	*p*-value	sHR (95% CI)	*p*-value
Age	1.03 (1.01–1.05)	**0** **.** **014**	1.00 (0.97–1.03)	0.99
Sex, M (vs. F)	1.11 (0.70–1.77)	0.67	–	
Smoking, pack-years	1.01 (1.00–1.01)	0.09	–	
Hypertension	3.61 (2.05–6.36)	**<0**.**001**	2.84 (1.54–5.23)	**0**.**001**
Hyperlipidemia	1.19 (0.75–1.90)	0.46	–	
Diabetes	2.09 (1.21–3.59)	**0**.**008**	1.20 (0.66–2.16)	0.55
Arrhythmia	2.09 (1.20–3.64)	**0**.**009**	1.17 (0.61–2.26)	0.63
CHF	4.04 (2.33–6.99)	**<0**.**001**	^	
CHD	3.68 (2.26–6.02)	**<0**.**001**	5.98 (2.99–11.94)	**<0**.**001**
Surgery	0.95 (0.58–1.55)	0.83	–	
Chemotherapy	1.01 (0.37–2.78)	0.98	–	
3D-CRT (vs. IMRT)	2.63 (1.20–5.88)	**0**.**015**	3.22 (1.42–7.14)	**0**.**005**
RAVI	1.02 (1.01–1.04)	**0**.**001**	1.02 (1.00–1.04)	**0**.**027**
Lung V55Gy	0.99 (0.95–1.04)	0.80	–	
Heart volume	1.01 (1.00–1.01)	**<0**.**001**	#	
LAVI	1.02 (1.01–1.03)	**0**.**001**	1.00 (0.98–1.02)	0.94
LCx V15Gy	1.01 (1.00–1.01)	**0**.**008**	#	
Lung D_max_	1.01 (0.98–1.04)	0.54	–	
Lung volume	1.00 (1.00–1.00)	0.47	–	
LM volume	3.63 (1.75–7.54)	**0**.**001**	#	
LAD V15Gy	1.01 (1.00–1.02)	**0**.**003**	1.03 (1.02–1.04)	**<0**.**001**
LM D_min_	1.01 (1.00–1.02)	0.10	–	
Esophagus V45Gy	1.01 (1.00–1.02)	0.20	–	
Interaction terms^a^				
CHD × RAVI	1.02 (0.99–1.06)	0.22	–	
CHD × heart volume	1.00 (0.99–1.01)	0.74	–	
CHD × LAVI	1.00 (0.95–1.04)	0.93	–	
CHD × LCx V15Gy	0.98 (0.97–0.99)	**0**.**003**	#	
CHD × LM volume	0.59 (0.13–2.80)	0.51	–	
CHD × LAD V15Gy	0.97 (0.96–0.99)	**<0**.**001**	0.97 (0.96–0.98)	**<0**.**001**

M, male; F, female; CHF, congestive heart failure; CHD, coronary heart disease; 3D-CRT, three-dimensional conformal radiation therapy; IMRT, intensity-modulated radiation therapy; RAVI, right atrial volume index; Gy, Gray; V55Gy, volume receiving 55 Gray; LAVI, left atrial volume index; D_max_, dose maximum; V15Gy, volume receiving 15 Gray; D_min_, dose minimum; V45Gy, volume receiving 45 Gray; LCx, left circumflex artery.

Bold indicates *p* < 0.05.

^a^ Interaction term between CHD as a dichotomous variable and ML-identified dose/volume or volume continuous variables (significant on univariable analysis).

^ Variable omitted due to being included within CHD variable.

# Variable omitted due to collinearity and overfitting.

After adjusting for age and ML-identified cardiovascular and cancer treatment factors, we observed similar results; each unit increase in RAVI was associated with a 2% increased risk of MACE (sHR 1.02, 95% CI: 1.01–1.04; *p* = 0.03). Among the baseline characteristics, CHD (sHR 5.98, 95% CI: 2.99–11.94; *p* < 0.001) and hypertension (sHR 2.84, 95% CI: 1.54–5.23; *p* = 0.001) significantly increased the risk of MACE. Among the RT and anatomical covariates, LADV15Gy (sHR 1.03, 95% CI: 1.02–1.04; *p* < 0.001) and utilization of 3D-CRT (vs. IMRT) (sHR 3.22, 95% CI: 1.42–7.14; *p* = 0.005) significantly increased risk of MACE (Table 2). LCxV15Gy also ranked highly but was excluded from the primary multivariable model due to its multicollinearity with LADV15Gy. A model substituting LADV15Gy with LCxV15Gy showed a significant relationship between MACE and RAVI (Supplementary Table S3). Given the collinearity between RAVI and LAVI, a separate analysis was performed using identical prognostic factors, which showed a significant relationship between LAVI and MACE. Each unit increase in LAVI was associated with a 1% increased risk of MACE (sHR 1.01, 95% CI: 1.00–1.03; *p* = 0.044), Supplementary Table S4.

### External validation of RAVI and LAVI

With a median follow-up of 4.5 years (IQR, 2.3–8.0 years) in the NSCLC subset (*n* = 102) and 6.5 years (IQR, 5.1–7.7) in the breast cancer subset (*n* = 181), there were 26 cases of MACE (*n* = 11 NSCLC, *n* = 15 breast cancer). The median time to MACE was 1.8 years in the lung cohort (IQR, 1.3–4.3 years) and 5.7 years in the breast cancer cohort (IQR, 4.4–7.1 years). The median RAVI was 43.7 ml/m^2^ (IQR, 35.9–54.9 ml/m^2^) and 42.6 ml/m^2^ (IQR, 35.2–49.0 ml/m^2^) for the NSCLC and breast cancer subsets, respectively. The median LAVI was 41.9 ml/m^2^ (IQR, 35.4–51.9 ml/m^2^) and 40.9 ml/m^2^ (IQR, 34.9–48.0 ml/m^2^) for the NSCLC and breast cancer subsets, respectively. The 2-year cumulative incidence of MACE in the NSCLC subset was 10.3% (95% CI, 5.3%–17.3%). The 5-year cumulative incidence in the breast cancer subset was 7.3% (95% CI, 4.0%–12.0%). Stratifying by quartiles, the cumulative incidence was greatest among the highest RAVI quartile (≥49.9 ml/m^2^; *p* = 0.02) and highest LAVI quartile (≥49.6 ml/m^2^; *p* < 0.001) (Figures 3C,D).

After adjusting for age and baseline CHD, RAVI was associated with an increased risk of MACE (lung cancer subset: sHR 1.05, 95% CI: 1.00–1.11, *p* = 0.044; breast cancer subset, sHR 1.06, 95% CI 1.01–1.11; *p* = 0.025; Table 3). There was a significant interaction between CHD and RAVI in the breast cancer cohort (*p* = 0.041), such that the risk of MACE associated with RAVI was more pronounced in those without baseline CHD (sHR 1.07, 95% CI: 1.02–1.12; *p* = 0.009) than those with CHD (sHR 0.96, 95% CI: 0.86–1.06; *p* = 0.36). LAVI showed similar findings in the breast cancer and NSCLC subsets (Supplementary Table S5), but no significant interaction between CHD and LAVI was observed.

**Table 3:** Multivariable Fine–Gray regression to predict MACE in external lung and breast cancer validation cohorts.

Covariable	Lung cancer^c^		Breast cancer^c^	
	sHR (95% CI)	*p*-value	sHR (95% CI)	*p*-value
Age	1.00 (0.94–1.06)	0.98	1.06 (0.97–1.15)	0.20
Sex (M vs. F)	1.90 (0.45–8.05)	0.38	Omitted	
Baseline CHD	6.56 (0.08–536.76)	0.40	402.90 (5.06–32,083.61)	0.007
RAVI^a^	1.05 (1.00–1.11)	0.044	1.06 (1.01–1.11)	0.025
CHD × RAVI^b^	0.98 (0.90–1.06)	0.63	0.91 (0.84–0.99)	0.041

sHR, subdistribution hazard ratio; CHD, coronary heart disease; RAVI, right atrial volume index.

^a^ Continuous variable (ml/m^2^).

^b^ Interaction term between CHD as a dichotomous variable and RAVI as a continuous variable.

^c^ Of *n* = 94 (lung) and *n* = 178 (breast) with BSA available for volume normalization.

## Discussion

In this multi-institutional retrospective study of nearly 1,000 patients with detailed cardiovascular and individual radiotherapy parameters, we utilized ML to analyze high-dimensionality clinical, anatomical, and dosimetric data. This allowed a less biased search through feature spaces to identify the most salient predictors. These data highlight readily available cardiovascular prognostic information acquired during radiation oncology point-of-care (routine CTs for RT planning). We report modest model performance for MACE prediction. Our ML framework not only identified risk factors consistent with previously reported (2) but also identified right and left atrial enlargement (RAVI, LAVI) as novel predictors of MACE following chest radiotherapy. Among these predictors, RAVI influences cardiac risk (24, 25) and ranked as the top predictive feature in our discovery cohort. LAVI—a physiologically related variable with known cardiac significance (26–28)—also ranked highly. The predictive value of RAVI and LAVI were externally validated on a mixed cohort of NSCLC and breast cancer patients, suggesting that both may offer distinct predictive value for MACE following chest radiotherapy. Deploying RAVI and LAVI estimation alongside existing cardiovascular risk assessments could provide a powerful tool during early point-of-care interventions and long-term surveillance of cancer survivors.

To our knowledge, this is the first study to demonstrate the association of indexed CT-derived cardiac chamber volumes with MACE following thoracic RT. In a recent study, Walls et al. (29) reported the association of left atrial volume with atrial arrhythmias utilizing the Northern Ireland Cardiovascular Health Events After Radiation Therapy (NI-HEART) study. However, their study did not index cardiac volumes to body habitus—an important distinction—since cardiac geometric dimensions vary by sex, body habitus, fitness, age, and ethnicity (30). Indeed, standard echocardiography practice involves indexing chamber volumes to body habitus (commonly BSA) and adjusting for sex and age (31). Furthermore, we utilized a single composite endpoint, the American Heart Association/American College of Cardiology-defined standard five-point MACE (19), which does not include atrial arrhythmias, whereas Walls et al. defined MACE as arrhythmias, acute HF, and myocardial infarction. These methodological differences and our larger cohort may explain our observed association between RAVI and LAVI with “MACE” compared with Walls et al.

Our findings are supported by several studies demonstrating the prognostic utility of atrial volume indices across the cardiovascular risk spectrum. Left atrial enlargement typically represents sequelae of chronic exposure to elevated cardiac filling pressures and is a well-established cardiac risk factor (23, 32). While classically evaluated from echocardiography, CT-derived LAVI is similarly associated with the risk of acute coronary syndrome (33). AI-based LAVI from lung cancer screening and coronary artery calcium CTs are associated with the risk of atrial fibrillation and MACE (34, 35). Fewer studies focus on RA enlargement, but a pathophysiological explanation for RAVI's pertinence in lung cancer could be the increased prevalence of comorbid cardiopulmonary diseases—obstructive and interstitial lung diseases—that drive increased right heart pressures (36–38). Growing research associates elevated RAVI with cardiac events, as RA remodeling is linked to arrhythmias and diastolic dysfunction (22, 39). RAVI may model risk conveyed by underlying cardiopulmonary disease given the potential for increased right heart pathology in these patients.

Given that multiple thoracic cancers demonstrate an increased risk of MACE after chest RT (3, 40–42), we evaluated the generalizability of RAVI and LAVI for MACE prediction by including both NSCLC and breast cancer patients during external validation. Among primary chest malignancies, posttreatment MACE rates are generally highest for lung cancer and lowest for breast cancer (6). Including both extremes suggests our results may generalize across broad baseline risks and cardiac radiation dose exposures. Moreover, patient populations and treatment paradigms for lung and breast cancer vary greatly, and our results may inform cardiovascular risk prediction across cancer histology and cardiovascular risk spectrums. Notably, new-onset HF and arrhythmias are increased within the first decade following breast RT (43, 44), possibly partially reflecting anthracycline exposure (45). Elevated RAVI/LAVI are plausible predictors for cardiac events in breast cancer given the mechanisms of treatment-related cardiotoxicity. Additional thoracic primaries demonstrate elevated MACE rates and share features with patients in this study. Lymphoma patients often receive multiple cycles of anthracyclines and consolidative thoracic RT (46). Risk factors for esophageal cancer overlap with patients with NSCLC and CHD, and these patients have elevated rates of concomitant cardiovascular disease and RT-related cardiac events (47). While our validation did not include all thoracic primaries, the shared risk and treatment factors suggest RAVI/LAVI could be applicable for risk stratification independent of cancer histology and is worthy of further investigation.

While typically quantified with echocardiograms in cardiovascular studies, atrial volumes estimated by CT are validated against echocardiograms (48–50). In the discovery cohort, median values for RAVI and LAVI were 51 ml/m^2^ and 48 ml/m^2^, respectively, compared with respective echocardiogram median estimates of 21 ml/m^2^ and 25 ml/m^2^ in healthy individuals (51, 52). Echocardiogram-based cutoffs of 35 ml/m^2^ for RAVI and 33–36 ml/m^2^ for LAVI show discriminatory power for cardiac dysfunction (53, 54). Standard-of-care chest or RT planning CTs lack cardiac gating and cannot fully account for dynamic changes in cardiac chamber volume when compared with ECG-gated CTs or echocardiograms. Measurement differences between these modalities may not translate to clinically meaningful discrepancies (50, 55), and cardiac chamber estimation via diagnostic CT appears feasible and relatively reproducible (56). Without established reference values for CT-based cardiac volume assessments, direct comparison of individual measurements is limited, and further investigation of specific cutoff values reflecting diverse cohorts would assist in translating these findings into clinical practice.

Studies consistently demonstrate underutilization of cardiac screening and medical optimization for cancer patients. For the NSCLC cohort, only half of the statin-eligible patients are on therapy (57), despite statins potentially decreasing the risk conveyed by higher heart RT dose and conferring a dose–response relationship with survival (58). Cardio-oncology guidelines recommend consideration of echocardiographic screening in patients with underlying cardiovascular disease before thoracic RT (59), but only 33% of our discovery cohort received an echocardiogram before RT. It is unclear if modern practice patterns are improved. Moreover, given the broad definitions and gaps in stratification for defining patients at high cardiovascular risk from RT in consensus guidelines, multiple studies have explored strategies to enhance upfront cardiovascular risk stratification. For instance, CT-based coronary artery calcification quantification shows promise in predicting MACE and mortality after chest RT (60–63). RAVI and LAVI show potential as radiologic markers to further inform CT-based risk stratification approaches. Our results support consensus guidelines that consider baseline echocardiographic screening. Advances in artificial intelligence-based approaches for automated segmentation of cardiac substructures (64–66) will provide opportunities for automation of RAVI and LAVI measurements on CT scans obtained at multiple time points during cancer care.

This study has limitations. Its retrospective nature is subject to sampling bias, misclassification, and follow-up bias. We recognize that systematic sampling bias cannot be fully accounted for. We believe the effect of follow-up bias on differences in observed rates of MACE to be low given the overall shorter time to MACE and relatively longer median follow-up within our cohort of patients entering longitudinal, routine cancer surveillance. Low MACE numbers within our validation cohort may increase the overfitting of our validation models. The concordance between estimates for RAVI/LAVI between discovery and validation modeling suggests that overfitting and bias were limited. Without established reference values for CT-based volumetric cardiac measurements and a lack of dynamic heart imaging, we were unable to assess atrial remodeling severity. Future work correlating CT-based atrial volume estimates with echocardiogram data and heart function, including analysis of atrial volume changes over time, is of interest. Our validation cohort was heterogenous compared with the discovery cohort, particularly with respect to treatment years and cancer type (inclusion of breast cancer in addition to NSCLC), but this could be considered a strength, since RAVI and LAVI remain significant predictors across time, primary tumor, and changes in cancer-directed therapies. Lastly, while the use of statins was included as a predictor, it was not ranked highly, and further studies should explore the impact of additional cardioprotective medications, such as beta-blockers, angiotensin-converting enzyme (ACE) inhibitors, and angiotensin receptor blockers (ARBs).

Our study utilized ML to analyze high-dimensionality clinical, anatomical, and dosimetric data, identifying novel predictors of MACE and externally validating on a mixed cohort of NSCLC and breast cancer patients. Elevated RAVI and LAVI may convey a higher risk for MACE following chest radiotherapy. Overall, deploying RAVI and LAVI estimation alongside existing cardiovascular risk assessments could provide a powerful tool during early point-of-care interventions and long-term surveillance of cancer survivors. The utility of RAVI and LAVI for the identification of high-risk patients warrants further study in prospective trials.

## Data Availability

The datasets presented in this article are not readily available because of institutional review board restrictions regarding human subject research. The data that support the findings of this study are available upon reasonable by a qualified investigator under a data use agreement and with appropriate ethical oversight. Requests to access the datasets should be directed to katelyn.atkins@cshs.org.
