# Supplementary material for: Baseline atrial volume indices and major adverse cardiac events following thoracic radiotherapy
Source: Front Cardiovasc Med. 2025 Jun 3;12:1560922. doi: 10.3389/fcvm.2025.1560922 (PMC12170593; doi:10.3389/fcvm.2025.1560922)
Supplement: Supplementary file 1 [file Datasheet1.pdf]

# *Supplementary Material*

**Supplementary Table 1.** Baseline characteristics of the discovery cohort, stratified by training and testing cohorts for initial machine learning discovery phase.

| Characteristics | Training (n=490) | Testing (n=211) | p |
|---|---|---|---|
| Age, median (IQR, years) | 65 (57, 73) | 65 (59, 72) | 0.93 |
| Female sex | 254 (51.8%) | 91 (43.1%) | 0.03 |
| Tobacco | | | |
| Never | 195 (39.8%) | 84 (39.8%) | |
| Current | 254 (51.8%) | 112 (53.0%) | |
| Former | 41 (8.4%) | 15 (7.2%) | 0.20 |
| Medical history | | | |
| HTN | 250 (51.0%) | 113 (53.6%) | 0.53 |
| HLD | 235 (47.6%) | 106 (50.2%) | 0.58 |
| DM | 66 (13.4%) | 31 (14.7%) | 0.67 |
| Stroke | 9 (1.8%) | 4 (1.9%) | 0.96 |
| CAD | 139 (28.4%) | 63 (29.9%) | 0.69 |
| CHF | 39 (7.9%) | 19 (9.0%) | 0.64 |
| Any CHD[#] | 171 (34.9%) | 81 (38.4%) | 0.38 |
| NSCLC clinical stage | | | |
| II | 53 (10.8%) | 26 (12.3%) | |
| III | 473 (89.2%) | 185 (87.7%) | 0.65 |
| Tumor laterality | | | |
| Right | 306 (62.4%) | 128 (60.7%) | 0.72 |
| Left | 184 (37.6%) | 83 (39.3%) | |
| Treatment | | | |
| Definitive CRT | 292 (59.6%) | 113 (53.6%) | |
| RT alone | 36 (7.3%) | 20 (9.5%) | |
| Neoadjuvant | 104 (21.2%) | 50 (23.7%) | |
| Adjuvant | 58 (11.8%) | 28 (13.3%) | 0.49 |
| RT technique | | | |
| 3D-CRT | 374 (76.3%) | 165 (78.2%) | 0.66 |
| IMRT | 116 (23.7%) | 46 (21.8%) | |
| RT dose | | | |
| Median (IQR, Gy) | 64.0 (54.0, 66.0) | 66.0 (60.0, 66.0) | 0.08 |

[#]CHD includes CAD, CHF, or CHD risk equivalent (stroke, peripheral artery disease).
Abbreviations: IQR, interquartile range; HTN, hypertension; HLD, hyperlipidemia; DM, diabetes mellitus; CAD, coronary artery disease; CHF, congestive heart failure; CHD, coronary heart disease; NSCLC, non-small cell lung cancer; CRT, chemoradiotherapy; RT, radiotherapy; 3D-CRT, 3-dimensional conformal radiotherapy; IMRT, intensity modulated radiotherapy.

**Supplementary Table 2. Additional Clinical Characteristics for Breast Validation Cohort**

| | Total (n=181) |
|---|---|
| ECOG PS | |
| 0 | 141 (79.2%) |
| 1 | 35 (19.7%) |
| 2 | 2 (1.1%) |
| Body mass index | 26 (23, 30) |
| Pathologic Stage (including yp) | |
| 0 | 25 (13.8%) |
| I | 54 (29.8%) |
| II | 71 (39.2%) |
| III | 27 (14.9%) |
| IV | 4 (2.2%) |
| Laterality | |
| Left | 77 (42.5%) |
| Right | 97 (53.6%) |
| Bilateral | 7 (3.9%) |
| Surgery | |
| Breast-conserving | 122 (67.4%) |
| Mastectomy | 53 (29.3%) |
| Systemic therapy | |
| Hormonal therapy | 133 (73.4%) |
| Any chemotherapy | 93 (51.4%) |
| Anthracycline | 35 (19.3%) |
| Her2 therapy | 27 (14.9%) |
| RT technique | |
| 3D-CRT | 175 (96.7%) |
| IMRT | 6 (3.3%) |
| DIBH | 40 (22.1%) overall; 36 (43.9%) of left-sided |
| RT dose x fractions | |
| 180-200 cGy x 22-28 | 125 (69.1%) |
| 266-267 cGy x 16 | 51 (28.2%) |
| Other | 5 (2.8%) |
| RT boost | 127 (70.2%) |
| RT target | |
| Breast | 105 (58.0%) |
| Breast + regional LN | 50 (27.6%) |
| Breast + regional LN + IMC | 5 (2.8%) |
| Chest wall | 4 (2.2%) |
| Chest wall + regional LN | 14 (7.7%) |
| Chest wall + regional LN + IMC | 2 (1.1%) |
| Other | 1 (0.6%) |

Values are median (interquartile range) or n (%). Abbreviations: IQR, interquartile range; RT, radiotherapy; 3D-CRT, 3-dimensional radiotherapy; IMRT, intensity-modulated radiotherapy; DIBH, deep inspiration breath hold; LN, lymph nodes; IMC, internal mammary chain.

| **Supplementary Table 3.** Fine and Gray regression model to predict MACE in discovery cohort, including left circumflex (instead of left anterior descending) coronary artery V15 Gy. | | | | |
|---|---|---|---|---|
| Covariable | Univariable | | multivariable | |
| | HR (95% CI) | p-value | HR (95% CI) | p-value |
| Age | 1.03 (1.01-1.05) | **0.014** | 1.00 (0.97-1.03) | 0.94 |
| Sex, M (vs F) | 1.11 (0.70-1.77) | 0.67 | - | |
| Smoking, pack years | 1.01 (1.00-1.01) | 0.09 | - | |
| Hypertension | 3.61 (2.05-6.36) | **<0.001** | 2.82 (1.55-5.13) | **0.001** |
| Hyperlipidemia | 1.19 (0.75-1.90) | 0.46 | - | |
| Diabetes | 2.09 (1.21-3.59) | **0.008** | 1.18 (0.67-2.10) | 0.57 |
| Arrhythmia | 2.09 (1.20-3.64) | **0.009** | 1.15 (0.59-2.24) | 0.67 |
| CHF | 4.04 (2.33-6.99) | **<0.001** | # | |
| CHD | 3.68 (2.26-6.02) | **<0.001** | 4.58 (2.32-9.01) | **<0.001** |
| Surgery | 0.95 (0.58-1.55) | 0.83 | - | |
| Chemotherapy | 1.01 (0.37-2.78) | 0.98 | - | |
| 3D-CRT (vs IMRT) | 2.63 (1.20-5.88) | **0.015** | 3.13 (1.41-7.14) | **0.006** |
| RAVI | 1.02 (1.01-1.04) | **0.001** | 1.02 (1.00-1.04) | **0.027** |
| Lung V55Gy | 0.99 (0.95-1.04) | 0.80 | - | |
| Heart volume | 1.01 (1.00-1.01) | **<0.001** | # | |
| LAVI | 1.02 (1.01-1.03) | **0.001** | 1.00 (0.98-1.02) | >0.99 |
| LCx V15Gy | 1.01 (1.00-1.01) | **0.008** | 1.02 (1.01-1.03) | **<0.001** |
| Lung $D_{max}$ | 1.01 (0.98-1.04) | 0.54 | - | |
| Lung volume | 1.00 (1.00-1.00) | 0.47 | - | |
| LM volume | 3.63 (1.75-7.54) | **0.001** | # | |
| LAD V15Gy | 1.01 (1.00-1.02) | **0.003** | # | |
| LM $D_{min}$ | 1.01 (1.00-1.02) | 0.10 | - | |
| Esophagus V45Gy | 1.01 (1.00-1.02) | 0.20 | - | |
| Interaction terms* | | | | |
| CHD:RAVI | 1.02 (0.99-1.06) | 0.22 | - | |
| CHD:Heart volume | 1.00 (0.99-1.01) | 0.74 | - | |
| CHD:LAVI | 1.00 (0.95-1.04) | 0.93 | - | |
| CHD:LCx V15Gy | 0.98 (0.97-0.99) | 0.003 | 0.97 (0.97-0.99) | **<.001** |
| CHD:LM volume | 0.59 (0.13-2.80) | 0.51 | - | |
| CHD:LAD V15Gy | 0.97 (0.96-0.99) | <0.001 | # | |

Abbreviations:
M, male; F, female; CHF, congestive heart failure; CHD, coronary heart disease; 3D-CRT, 3-dimensional conformal radiation therapy; IMRT, intensity modulated radiation therapy; RAVI, right atrial volume index; Gy, Gray; V55Gy, volume receiving 55 Gray; LAVI, left atrial volume index; $D_{max}$, dose maximum; V15Gy, volume receiving 15 Gray; $D_{min}$, dose minimum; V45Gy, volume receiving 45 Gray; LCx, left circumflex artery.

*Interaction term between CHD as dichotomous variable and ML-identified dose-volume or volume continuous variables (significant on univariable analysis). ^variable omitted due to being included within CHD variable. #variable omitted due to collinearity and over-fitting.

**Supplementary Table 4.** Fine and Gray regression model to predict MACE in the discovery cohort with left atrial volume index (LAVI) compared with right atrial volume index (RAVI)

| Covariable | LAVI model | | RAVI model | |
|---|---|---|---|---|
| | HR (95% CI) | p-value | HR (95% CI) | p-value |
| Age | 1.00 (0.97-1.02) | 0.83 | 1.00 (0.97-1.03) | 0.94 |
| Sex, M (vs F) | 0.80 (0.49-1.31) | 0.38 | 0.77 (0.47-1.27) | 0.31 |
| Hypertension | 2.94 (1.59-5.43) | 0.001 | 2.97 (1.55-5.34) | 0.001 |
| Diabetes | 1.21 (0.67-2.18) | 0.52 | 1.22 (0.68-2.23) | 0.50 |
| Arrythmias | 1.34 (0.70-2.18) | 0.88 | 1.19 (0.62-2.31) | 0.53 |
| CHD | 5.58 (2.82-11.07) | <0.001 | 5.95 (2.98-11.89) | <0.001 |
| 3D-CRT (vs IMRT) | 2.63 (1.19-5.88) | .017 | 3.33 (1.47-7.69) | 0.004 |
| LAVI | 1.01 (1.00-1.03) | .044 | # | # |
| RAVI | # | # | 1.02 (1.01-1.04) | 0.005 |
| LAD V15Gy | 1.03 (1.02-1.04) | <0.001 | 1.03 (1.02-1.04) | <0.001 |
| Interaction terms* CHD:LADV15 | 0.97 (0.96-0.99) | <0.001 | 0.97 (0.96-0.99) | <0.001 |

#Omitted
Abbreviations:
M, male; F, female; CHD, coronary heart disease; 3D-CRT, 3-dimensional conformal radiation therapy; IMRT, intensity modulated radiation therapy; Gy, Gray; V15Gy, volume receiving 15 Gray; LAD, left anterior descending artery

**Supplementary Table 5.** Multivariable Fine and Gray regression to predict MACE in external lung and breast cancer validation cohorts.

| Covariable | Lung cancer† | | Breast cancer† | |
|---|---|---|---|---|
| | sHR (95% CI) | p-value | sHR (95% CI) | p-value |
| Age | 0.99 (0.94-1.05) | 0.73 | 1.03 (0.95-1.12) | 0.46 |
| Sex (M vs F) | 1.94 (0.42-8.88) | 0.39 | omitted | |
| Baseline CHD | 18.04 (0.16-2.10x$10^2$) | 0.23 | 37.6 (1.03-1.37x$10^3$) | 0.048 |
| LAVI* | 1.09 (1.00-1.17) | 0.040 | 1.05 (1.02-1.08) | 0.001 |
| CHD x LAVI# | 0.96 (0.88-1.04) | 0.29 | 0.96 (0.92-1.01) | 0.144 |

*Continuous variable (mL/$m^2$). Abbreviations: sHR, subdistribution hazard ratio; CHD, coronary heart disease; RAVI, right atrial volume index.

#Interaction term between CHD as dichotomous variable and RAVI as continuous variable.

†Of n=94 (lung) and n=178 (breast) with BSA available for volume normalization.

**Appendix**
List of all features used for initial machine learning algorithm.

Body surface area

Age

Gender - Female

Performance status

Weight loss

Active smoker

Former smoker

Never smoker

Clinical stage IIA

Clinical stage IIB

Clinical stage IIIA

Clinical stage IIIB

Clinical T0

Clinical T1a

Clinical T1b

Clinical T2a

Clinical T2b

Clinical T3

Clinical T4

Clinical Tx

Clinical N0

Clinical N1

Clinical N2

Clinical N3

Clinical Nx

Tumor location - Left vs Right lobe

Tumor location - Left endobronchial

Tumor location - Left lower lobe

Tumor location - Left upper lobe

Tumor location - Right endobronchial

Tumor location - Right Lower Lobe

Tumor location - Right middle lobe

Tumor location - Right upper lobe

Tumor location - Adenocarcinoma

History of hypertension

History of hyperlipidemia

Diabetes Mellitus

History of stroke

History of myocardial infarction

History of congestive heart failure

History of Chronic obstructive pulmonary disease

Chemotherapy use yes/no

Tumor Size

Number of lymph node stations

Elective nodal irradiation

Concurrent chemotherapy yes/no

Concurrent chemotherapy with Alimta

Concurrent chemotherapy with Carboplatin/Alimta (Pemetrexed)

Concurrent chemotherapy with Carboplatin/Taxol/Avastin

Concurrent chemotherapy with Cisplatin/Alimta (Pemetrexed)

Concurrent chemotherapy with Cisplatin/Etoposide (EP5050)

Concurrent chemotherapy with Cisplatin/Irinotecan

Concurrent chemotherapy with Cisplatin/Navelbine

Concurrent chemotherapy with Cisplatin/Taxotere

Concurrent chemotherapy with Navelbine

Concurrent chemotherapy unknown chemotherapy

Concurrent chemotherapy with other chemotherapy

Concurrent chemotherapy with Carboplatin/Etoposide

Concurrent chemotherapy with Carboplatin/Paclitaxel (Taxol)

Concurrent chemotherapy with Cisplatin/Etoposide every 3 weeks

Concurrent chemotherapy with Tarceva

Concurrent chemotherapy with Carboplatin/Taxol weekly

Concurrent chemotherapy with carboplatin/taxotere weekly

Number of cycles of concurrent chemotherapy

Smoking pack years

Prior lung surgery

Prior thoracic radiation

Coronary calcifications on CT without formal diagnosis

Recurrence of tumor

Pancoast tumor

Treatment intent - definitive

Treatment intent - Neoadjuvant or adjuvant

Induction chemotherapy yes/no

Number of cycles of induction chemotherapy

Induction chemotherapy with Carboplatin/Alimta (Pemetrexed)

Induction chemotherapy with Carboplatin/Alimta/Avastin

Induction chemotherapy with Carboplatin/Gemcitabine

Induction chemotherapy with Carboplatin/Taxol/Avastin

Induction chemotherapy with Carboplatin/Taxotere

Induction chemotherapy with Cisplatin/Alimta (Pemetrexed)

Induction chemotherapy with Cisplatin/Etoposide (EP5050)

Induction chemotherapy with Cisplatin/Navelbine

Induction chemotherapy with Cisplatin/Taxotere

Induction chemotherapy with Gemcitabine/Cisplatin

Induction chemotherapy with Navelbine

Induction chemotherapy with unknown agent

Induction chemotherapy with Other chemotherapy

Induction chemotherapy with Carboplatin/Etoposide every 3 weeks

Induction chemotherapy with Carboplatin/Paclitaxel (Taxol) every 3 weeks

Induction chemotherapy with Cisplatin/Etoposide every 3 weeks

Induction chemotherapy with Tarceva

Induction chemotherapy with Carboplatin/Taxol weekly

Adjuvant chemotherapy yes/no

Adjuvant chemotherapy with Alimta

Adjuvant chemotherapy with Carboplatin/Alimta (Pemetrexed)

Adjuvant chemotherapy with Carboplatin/Gemcitabine

Adjuvant chemotherapy with Carboplatin/Taxol/Avastin

Adjuvant chemotherapy with Carboplatin/Taxotere

Adjuvant chemotherapy with Cisplatin/Alimta (Pemetrexed)

Adjuvant chemotherapy with Cisplatin/Etoposide (EP5050)

Adjuvant chemotherapy with Cisplatin/Irinotecan

Adjuvant chemotherapy with Cisplatin/Navelbine

Adjuvant chemotherapy with Cisplatin/Taxotere

Adjuvant chemotherapy with Gemcitabine/Cisplatin

Adjuvant chemotherapy with Navelbine

Adjuvant chemotherapy with unknown agent

Adjuvant chemotherapy with other agent

Adjuvant chemotherapy with Carboplatin/Etoposide every 3 weeks

Adjuvant chemotherapy with Carboplatin/Paclitaxel (Taxol) every 3 weeks

Adjuvant chemotherapy with Cisplatin/Etoposide every 3 weeks

Adjuvant chemotherapy with Tarceva

Adjuvant chemotherapy with Taxol

Adjuvant chemotherapy with Taxotere

Adjuvant chemotherapy with Carboplatin/Taxol weekly

Number of cycles of chemotherapy

History of coronary heart disease

History of hypertension

On statin

Lymph node station involved - Left Hilar

Lymph node station involved - Right Supraclavicular

Lymph node station involved - Left Supraclavicular

Lymph node station involved - Right upper paratracheal

Lymph node station involved - Left upper paratracheal

Lymph node station involved - Prevascular

Lymph node station involved - Retrotracheal

Lymph node station involved - Right lower paratracheal

Lymph node station involved - Left lower paratracheal

Lymph node station involved - Subaortic AP Window

Lymph node station involved - Paraaortic ascending or phrenic

Lymph node station involved - Subcarinal

Lymph node station involved - Paraesophageal below carina

Lymph node station involved - Pulmonary ligament

Lymph node station involved - Right Hilar

Unknown lymph node station

GTV includes Hilar and mediastinal nodes

GTV includes Hilar nodes

GTV includes Hilar, mediastinal + supraclavicular nodes

GGTV includes Mediastinal + supraclavicular nodes

GTV includes Mediastinal nodes

GTV includes unknown

GTV includes Primary tumor

GTV includes Primary tumor + hilar + mediastinal + supraclavicular lymph nodes

GTV includes Primary tumor + hilar + mediastinal lymph nodes

GTV includes Primary tumor + hilar lymph nodes

GTV includes Primary tumor + mediastinal lymph nodes

GTV includes Primary tumor + mediastinal lymph nodes+ supraclavicular lymph nodes

GTV includes Primary tumor + supraclavicular lymph nodes

Elective Nodal Irradiation - Left Hilar

Elective Nodal Irradiation - Hilar

Elective Nodal Irradiation - Left upper paratracheal

Elective Nodal Irradiation - Right upper paratracheal

Elective Nodal Irradiation - Prevascular

Elective Nodal Irradiation - Retrotracheal

Elective Nodal Irradiation - Left lower paratracheal

Elective Nodal Irradiation - Right lower paratracheal

Elective Nodal Irradiation - Subaortic AP Window

Elective Nodal Irradiation - Ascending aortic or phrenic

Elective Nodal Irradiation - Subcarinal

Elective Nodal Irradiation - Paraesophageal below carina

Elective Nodal Irradiation - Pulmonary ligament

Elective Nodal Irradiation - Left Supraclavicular

Elective Nodal Irradiation - None

Elective Nodal Irradiation - Right Supraclavicular

Treatment intent - Adjuvant

Treatment intent - Palliative

Treatment intent - Preoperative

Treatment intent - Preoperative and adjuvant

Treatment intent - Radical (definitive)

History of coronary artery disease

Not on anticoagulation at diagnosis

CT sim with contrast yes/no

IGRT use yes

IGRT use no

IGRT use unknown

3D simulation

4D simulation

Partial 4D simulation

Unknown CT simulation

History of diastolic dysfunction on echocardiogram

Prior cardiac echocardiogram

Home O2 use

History of arrythmia

History cardiac valvular disease

Coronary artery class 0 (2018)

Coronary artery class 1 (2018)

Coronary artery class 2 (2018)

Coronary artery class 3 (2018)

Coronary artery class unknown (2018)

Coronary artery class 0 (2019)

Coronary artery class 1 (2019)

Coronary artery class 2 (2019)

Coronary artery class 3 (2019)

Coronary artery class unknown (2019)

Biologically equivalent dose in 10 Gy

Major adverse cardiac event

Intensity modulated radiation therapy vs. 3D conformal radiation therapy

Coronary arteries (all) max dose

Coronary arteries (all) mean dose

Coronary arteries (all) min dose

Coronary arteries (all) V5 Gy

Coronary arteries (all) V10 Gy

Coronary arteries (all) V15 Gy

Coronary arteries (all) V20 Gy

Coronary arteries (all) V25 Gy

Coronary arteries (all) V30 Gy

Coronary arteries (all) V35 Gy

Coronary arteries (all) V40 Gy

Coronary arteries (all) V45 Gy

Coronary arteries (all) V50 Gy

Coronary arteries (all) V55 Gy

Coronary arteries (all) V60 Gy

Coronary arteries (all) total volume

Esophagus max dose

Esophagus mean dose

Esophagus min dose

Esophagus V5 Gy

Esophagus V10 Gy

Esophagus V15 Gy

Esophagus V20 Gy

Esophagus V25 Gy

Esophagus V30 Gy

Esophagus V35 Gy

Esophagus V40 Gy

Esophagus V45 Gy

Esophagus V50 Gy

Esophagus V55 Gy

Esophagus V60 Gy

Esophagus total volume

Heart max dose

Heart mean dose

Heart min dose

Heart V5 Gy

Heart V10 Gy

Heart V15 Gy

Heart V20 Gy

Heart V25 Gy

Heart V30 Gy

Heart V35 Gy

Heart V40 Gy

Heart V45 Gy

Heart V50 Gy

Heart V55 Gy

Heart V60 Gy

Heart total volume

Left atrium max dose

Left atrium mean dose

Left atrium min dose

Left atrium V5 Gy

Left atrium V10 Gy

Left atrium V15 Gy

Left atrium V20 Gy

Left atrium V25 Gy

Left atrium V30 Gy

Left atrium V35 Gy

Left atrium V40 Gy

Left atrium V45 Gy

Left atrium V50 Gy

Left atrium V55 Gy

Left atrium V60 Gy

Left atrium total volume

Left anterior descending coronary artery max dose

Left anterior descending coronary artery mean dose

Left anterior descending coronary artery min dose

Left anterior descending coronary artery V5 Gy

Left anterior descending coronary artery V10 Gy

Left anterior descending coronary artery V15 Gy

Left anterior descending coronary artery V20 Gy

Left anterior descending coronary artery V25 Gy

Left anterior descending coronary artery V30 Gy

Left anterior descending coronary artery V35 Gy

Left anterior descending coronary artery V40 Gy

Left anterior descending coronary artery V45 Gy

Left anterior descending coronary artery V50 Gy

Left anterior descending coronary artery V55 Gy

Left anterior descending coronary artery V60 Gy

Left anterior descending coronary artery total volume

Left circumflex coronary artery max dose

Left circumflex coronary artery mean dose

Left circumflex coronary artery min dose

Left circumflex coronary artery V5 Gy

Left circumflex coronary artery V10 Gy

Left circumflex coronary artery V15 Gy

Left circumflex coronary artery V20 Gy

Left circumflex coronary artery V25 Gy

Left circumflex coronary artery V30 Gy

Left circumflex coronary artery V35 Gy

Left circumflex coronary artery V40 Gy

Left circumflex coronary artery V45 Gy

Left circumflex coronary artery V50 Gy

Left circumflex coronary artery V55 Gy

Left circumflex coronary artery V60 Gy

Left circumflex coronary artery total volume

Left main coronary artery max dose

Left main coronary artery mean dose

Left main coronary artery min dose

Left main coronary artery V5 Gy

Left main coronary artery V10 Gy

Left main coronary artery V15 Gy

Left main coronary artery V20 Gy

Left main coronary artery V25 Gy

Left main coronary artery V30 Gy

Left main coronary artery V35 Gy

Left main coronary artery V40 Gy

Left main coronary artery V45 Gy

Left main coronary artery V50 Gy

Left main coronary artery V55 Gy

Left main coronary artery V60 Gy

Left main coronary artery total volume

Lung max dose

Lung mean dose

Lung min dose

Lung V5 Gy

Lung V10 Gy

Lung V15 Gy

Lung V20 Gy

Lung V25 Gy

Lung V30 Gy

Lung V35 Gy

Lung V40 Gy

Lung V45 Gy

Lung V50 Gy

Lung V55 Gy

Lung V60 Gy

Lung total volume

Left ventricle max dose

Left ventricle mean dose

Left ventricle min dose

Left ventricle V5 Gy

Left ventricle V10 Gy

Left ventricle V15 Gy

Left ventricle V20 Gy

Left ventricle V25 Gy

Left ventricle V30 Gy

Left ventricle V35 Gy

Left ventricle V40 Gy

Left ventricle V45 Gy

Left ventricle V50 Gy

Left ventricle V55 Gy

Left ventricle V60 Gy

Left ventricle total volume

Right atrium ventricle max dose

Right atrium ventricle mean dose

Right atrium ventricle min dose

Right atrium ventricle V5 Gy

Right atrium ventricle V10 Gy

Right atrium ventricle V15 Gy

Right atrium ventricle V20 Gy

Right atrium ventricle V25 Gy

Right atrium ventricle V30 Gy

Right atrium ventricle V35 Gy

Right atrium ventricle V40 Gy

Right atrium ventricle V45 Gy

Right atrium ventricle V50 Gy

Right atrium ventricle V55 Gy

Right atrium ventricle V60 Gy

Right atrium ventricle total volume

Right coronary artery max dose

Right coronary artery mean dose

Right coronary artery min dose

Right coronary artery V5 Gy

Right coronary artery V10 Gy

Right coronary artery V15 Gy

Right coronary artery V20 Gy

Right coronary artery V25 Gy

Right coronary artery V30 Gy

Right coronary artery V35 Gy

Right coronary artery V40 Gy

Right coronary artery V45 Gy

Right coronary artery V50 Gy

Right coronary artery V55 Gy

Right coronary artery V60 Gy

Right coronary artery total volume

Right ventricle max dose

Right ventricle mean dose

Right ventricle min dose

Right ventricle V5 Gy

Right ventricle V10 Gy

Right ventricle V15 Gy

Right ventricle V20 Gy

Right ventricle V25 Gy

Right ventricle V30 Gy

Right ventricle V35 Gy

Right ventricle V40 Gy

Right ventricle V45 Gy

Right ventricle V50 Gy

Right ventricle V55 Gy

Right ventricle V60 Gy

Right ventricle total volume
